# Incivility in COVID-19 Vaccine Mandate Discourse and Moral Foundations: Natural Language Processing Approach

**DOI:** 10.2196/50367

**Published:** 2023-11-29

**Authors:** Jason Tin, Hannah Stevens, Muhammad Ehab Rasul, Laramie D Taylor

**Affiliations:** 1 Department of Public Health Sciences University of California, Davis Davis, CA United States; 2 Department of Population and Quantitative Health Sciences University of Massachusetts Chan Medical School Worcester, MA United States; 3 Department of Communication University of California, Davis Davis, CA United States

**Keywords:** incivility, vaccine hesitancy, moral foundations, COVID-19, vaccines, morality, social media, natural language processing, machine learning

## Abstract

**Background:**

Vaccine hesitancy poses a substantial threat to efforts to mitigate the harmful effects of the COVID-19 pandemic. To combat vaccine hesitancy, officials in the United States issued vaccine mandates, which were met with strong antivaccine discourse on social media platforms such as Reddit. The politicized and polarized nature of COVID-19 on social media has fueled uncivil discourse related to vaccine mandates, which is known to decrease confidence in COVID-19 vaccines.

**Objective:**

This study examines the moral foundations underlying uncivil COVID-19 vaccine discourse. Moral foundations theory poses that individuals make decisions to express approval or disapproval (ie, uncivil discourse) based on innate moral values. We examine whether moral foundations are associated with dimensions of incivility. Further, we explore whether there are any differences in the presence of incivility between the r/coronaviruscirclejerk and r/lockdownskepticism subreddits.

**Methods:**

Natural language processing methodologies were leveraged to analyze the moral foundations underlying uncivil discourse in 2 prominent antivaccine subreddits, r/coronaviruscirclejerk and r/lockdownskepticism. All posts and comments from both of the subreddits were collected since their inception in March 2022. This was followed by filtering the data set for key terms associated with the COVID-19 vaccine (eg, “vaccinate” and “Pfizer”) and mandates (eg, “forced” and “mandating”). These key terms were selected based on a review of existing literature and because of their salience in both of the subreddits. A 10% sample of the filtered key terms was used for the final analysis.

**Results:**

Findings suggested that moral foundations play a role in the psychological processes underlying uncivil vaccine mandate discourse. Specifically, we found substantial associations between all moral foundations (ie, care and harm, fairness and cheating, loyalty and betrayal, authority and subversion, and sanctity and degradation) and dimensions of incivility (ie, toxicity, insults, profanity, threat, and identity attack) except for the authority foundation. We also found statistically significant differences between r/coronaviruscirclejerk and r/lockdownskepticism for the presence of the dimensions of incivility. Specifically, the mean of identity attack, insult, toxicity, profanity, and threat in the r/lockdownskepticism subreddit was significantly lower than that in the r/coronaviruscirclejerk subreddit (*P*<.001).

**Conclusions:**

This study shows that moral foundations may play a substantial role in the presence of incivility in vaccine discourse. On the basis of the findings of the study, public health practitioners should tailor messaging by addressing the moral values underlying the concerns people may have about vaccines, which could manifest as uncivil discourse. Another way to tailor public health messaging could be to direct it to parts of social media platforms with increased uncivil discourse. By integrating moral foundations, public health messaging may increase compliance and promote civil discourse surrounding COVID-19.

## Introduction

### Overview

As of October 2023, more than 6,959,316 deaths and 770,875,433 cases of COVID-19 have been reported worldwide [1]. Despite the mass availability of vaccines in the United States, 32.8% of the population remains unvaccinated [2]. To increase vaccination numbers, vaccine mandates were issued across the United States. While some US adults complied with the mandates, others reacted with incivility [3]. Many public officials have caused public outrage against COVID-19 vaccines by issuing statements that minimize vaccine efficacy [4]. As such, the politicized nature of COVID-19 has increased the salience of political ideology in public health discourse [5] and sparked negative sentiment toward vaccines, which may fuel incivility toward vaccines and mandates [6,7].

Public reliance on social media (eg, Reddit) increased heavily during the COVID-19 pandemic for various reasons, including seeking information to ease pandemic anxiety and due to social distancing—in addition to news consumption [8]. This gave rise to what scholars have coined an “infodemic” [9], where the unabated spread of COVID-19 misinformation on social media platforms undermined public trust in public health officials and their guidelines [10]. Recent work has shown that increased consumption of news related to COVID-19 leads to vaccine hesitancy and that engaging with the news on social media is linked to increased sharing and belief of COVID-19 misinformation due to various reasons, such as social media fatigue [11,12]. Uncivil vaccine discourse (eg, “Fucking Disneyland isn’t enforcing masks anymore there is no fucking reason for your college to do so especially since they are requiring the vaccine”) also decreases vaccine uptake [13,14]. Yet, the psychological mechanism underlying uncivil vaccine discourse remains unclear. Therefore, understanding the psychological processes underlying uncivil COVID-19 vaccine discourse on social media platforms is necessary to inform effective interventions.

Incivility has been investigated across various social media platforms in political and health contexts [15-19]. Although a few studies have linked negative emotions with incivility [20], more work is needed in this area to understand the psychological processes that prompt emotion-fueled uncivil discourse. Moreover, although scholars have extensively studied COVID-19 across various contexts [3,9-11,21], very few studies have attempted to identify the theoretical underpinnings of the discourse surrounding the virus. This work fills this gap by examining the moral foundations underlying uncivil discourses on 2 prominent antivaccine subreddits by using natural language processing techniques.

Moral foundations help individuals make decisions based on 5 innate moral values (care and harm, fairness and cheating, loyalty and betrayal, authority and subversion, and sanctity and degradation). Investigating the moral foundations of uncivil vaccine discourse can provide insight into the drivers of that incivility and offer practical implications for public health interventions against COVID-19. Thus, this work meaningfully contributes to the existing literature focused on eradicating the negative impact of COVID-19 through vaccine uptake.

### Incivility

#### Definition

Scholars across different fields have found it difficult to develop one definition of incivility. Some studies have defined incivility as impoliteness, profanity, or specific actions such as derogatory language used by political officials [16]. Coe et al [22] categorize incivility as using hateful, pejorative, or disrespectful language. Other studies have added to these definitions by including ideologically extreme arguments, exaggerated arguments, and misinformation as indicators of incivility [23-25]. Some cross-disciplinary fields conceptualize incivility as violations of norms of politeness, hostile interruptions, disrespectful behaviors, defensive reactions, and refusing to acknowledge opposing views [26-28]. We conceptualize incivility as a multidimensional construct, including toxicity, profanity, threats, insults, and discriminatory language [20].

#### Moral Foundations Theory

##### Overview

The moral foundations theory (MFT) offers one explanation for vaccine discourse incivility on social media platforms. The MFT posits that individuals can adaptively make decisions and express approval or disapproval based on 5 innate moral values: care and harm, fairness and cheating, loyalty and betrayal, authority and subversion, and sanctity and degradation [29]. The care and harm dimension points out the difference between protection and the mistreatment of individuals, whereas the fairness and cheating values highlight the contrast between impartiality and dishonesty. The loyalty and betrayal values involve intergroup attachment. The authority and subversion dimension refers to the degree to which an individual follows or opposes authority. Lastly, the sanctity and degradation values focus on spirituality. Threatening individuals’ moral values can provoke uncivil behavior, such as verbally attacking vaccine proponents, to express disapproval.

The MFT reasons that everyone shares the same core moral values. However, individuals prioritize moral values based on external factors such as cultural and environmental influences [30]. Indeed, the relative importance of individuals’ moral values is linked to compliance with COVID-19 protective measures [31-33]. An individual’s perceptions that their moral values are threatened can provoke uncivil behavior, such as verbally attacking vaccine proponents, to reassert those values; a recent study found a substantial relationship between incivility and moral foundations in social media discourse [34].

Although researchers have used surveys to investigate moral foundations and behavior during the COVID-19 pandemic [32], such as incivility toward Asians [34], self-report data insight is limited. This work identifies salient moral foundations underlying uncivil COVID-19 vaccine discourse in an observational setting on Reddit, a social media platform that contains uncivil discourse in a naturalistic setting. Reddit provides a valuable platform to study incivility in part because it is consumed (and contributed to) by individuals worldwide (eg, in 2015, over 200 million individuals visited Reddit from 208 countries) [35]. Reddit also allows researchers to observe specific subreddits composed of individuals from certain backgrounds [36]. Therefore, we propose the following research question (RQ):

- RQ1: What moral foundations are linked to uncivil COVID-19 discourse?

##### Sanctity and Degradation

The sanctity and degradation moral foundation refers to purity in both the spiritual and physical sense. People who value physical sanctity aim to preserve their bodily well-being. While some research has found the sanctity foundation to predict the usage of masks during the COVID-19 pandemic, other work has found that it can predict vaccine hesitancy [32,37]. One explanation is that individuals may perceive foreign substances (the vaccine, rather than a pathogen) as impure; in other words, individuals with opposing views may share a salient moral foundation [38].

Research suggests that purity is an area of political disagreement [39]. Underpinned by cognitive dissonance theory [40,41], vaccine proponents, who may believe in vaccinations to keep their body pure from the virus, may experience cognitive discomfort in discussion with vaccine opponents, who believe vaccinations are impure, and vice versa. Researchers have argued that issues driven by values of sanctity and degradation (ie, lesbian, gay, bisexual, transgender, queer [LGBTQ] issues) result in fierce opposition because it violates a sense of purity held by anti-LGBTQ individuals [42,43]. Similarly, vaccine proponents and opponents may act uncivilly and attack each other to protect their moral perception of purity. As such, we hypothesize that:

- H1: Purity will positively predict COVID-19 vaccine mandate incivility.

##### Individualizing Foundations (Care and Fairness)

The existing literature on MFT categorizes distinct moral foundations into two clusters: (1) individualizing foundations (ie, care and fairness), which concern the value of the individual, and (2) binding foundations (ie, loyalty, authority, and sanctity), which concern group integrity [44].

Individualizing moral foundations (ie, foundations care and harm, and fairness and cheating) are linked to protective COVID-19 behaviors [31,32]. Existing work suggests that individualizing foundations are the most relevant in moral decisions when faced with a disease threat [33]. Additionally, the polarized state of US politics may have contributed to uncivil COVID-19 discussion on social media platforms. Past research has shown that liberal individuals tend to value individualizing foundations, whereas conservative individuals tend to value binding foundations, though some studies have shown that conservatives may value all 5 foundations equally [44-48]. Additionally, scholars have pointed out that individuals who value individualizing foundations are more likely to respond emotionally to uncivil comments [49]. Liberal individuals may experience cognitive dissonance and stress when faced with antivaccine mandate messaging [40,41]. These individuals may be more likely to engage in uncivil behaviors and attack antivaccine mandate messaging promoters to reduce discomfort. Scholars have pointed out that individualizing foundations such as care and fairness are more important to liberals than conservatives, although these foundations are not limited to one political ideology [44]. Given the heavily politicized nature of COVID-19 [5], violating the care and fairness foundations may elicit COVID-19 vaccine discourse incivility. Therefore, we hypothesize that:

- H2: Care will positively predict COVID-19 vaccine mandate incivility, and
- H3: Fairness will positively predict COVID-19 vaccine mandate incivility.

##### Binding Foundations (Loyalty and Betrayal)

Loyalty and betrayal or in-group loyalty refers to a person’s allegiance and devotion to their own group, and it may also have a role in COVID-19 discourse incivility. The pandemic gave rise to 2 different groups, individuals who encouraged protective pandemic behaviors (eg, masking and vaccinating) and individuals who disregarded protective measures (eg, 1 study observed that non–mask wearers were more likely to cooperate with other non–mask wearers than mask wearers) [14], suggesting the influence of an in-group bias. Another study observed that COVID-19 discussion on social media platforms was politicized, with right-leaning users tending to engage less with health-promoting hashtags on Twitter (now known as X) than left-leaning users [3]. These findings are in conjunction with existing research, which argues that opinions about the COVID-19 pandemic and vaccine are split along partisan lines, with Republicans exhibiting negative attitudes toward the vaccine [50] and reporting lower intentions to get vaccinated due to increased misperceptions about side effects [51]. In turn, the polarization surrounding COVID-19 could have resulted in partisan-motivated reasoning, where individuals’ prior proattitudinal beliefs and their partisan alignment drive information processing [52]. As such, conservatives may have engaged in uncivil discourse against the COVID-19 vaccine, whereas liberals may have engaged in uncivil discourse in favor of the vaccine to ensure that their vaccine stance aligns with their prior political beliefs. On the basis of the overview of existing research above, we hypothesize that:

- H4: In-group loyalty will positively predict COVID-19 vaccine mandate incivility.

##### Authority and Subversion

Existing literature suggests that individuals condemn perceived leadership failures in hierarchical organizations [43]. Thus, authority and subversion foundations may incite incivility. Many protective pandemic guidelines have been created, supported, and sometimes enforced by authority figures and leaders (eg, locally elected politicians and the Centers for Disease Control and Prevention [CDC]). For example, right-wing outlets blasted the COVID-19 vaccine and exacerbated the spread of COVID-19 vaccine misinformation [53]. Additionally, Donald Trump, the US president at the time, continuously referred to COVID-19 as a hoax, whereas Democrats condemned politicians who minimized the pandemic’s threat and vaccine efficacy. Such condemnation may have manifested as uncivil comments directed at these government officials and public health authorities. Therefore, we argue that authority and subversion values can prompt uncivil discourse.

- H5: Authority will positively predict incivility in discussion about COVID-19 vaccine mandates.

#### Social Media Culture Norms

Organizational research suggests that incivility results from interactions between community and situational norms, which are shaped by organizational policies [17]. Cultural norms and platform capabilities can enable or mitigate incivility (ie, Twitter’s hateful conduct policy, Reddit’s moderation rules, and Facebook’s community standards) [54,55]. Social media platforms also respond to elements of incivility differently due to cultural norms, which can impact the frequency of uncivil interactions on the platforms. Indeed, Facebook is known to have less uncivil discourse than Twitter [56], but intolerant comments are more frequently found on Facebook [57]. In the context of our study, different subreddits across Reddit have various norms that are distinct from each other. For example, the subreddit r/lockdownskepticism has the following description of their rules that users have to agree to before joining: “Interdisciplinary examination of lockdowns & other pandemic policies. We acknowledge the threat of COVID-19. We are also concerned about the policies’ impact on our physical and mental health, human rights, and economy. This is a non-partisan, inclusive, global sub. We are empirically minded and do not tolerate unsupported claims or conspiracy theories. **Warning: users may be auto-banned from other subs for posting here**.” In contrast, the subreddit r/coronaviruscirclejerk has a description that starts with, “We are all going to die,” and is more focused on the memes and discussion resulting from the discourse between “panic-filled” individuals and “alarmists” on the web. This indicates some cultural norm differences that exist among subreddits on Reddit. Other social media sites may have different cultural norms that may impact incivility differently. Shmargad et al [58] point out that the frequency of incivility on platforms is dependent on platform norms (eg, moderator rules). On the basis of this discussion, we pose the following RQ:

- RQ2: Does discourse incivility vary by cultural norms?

## Methods

### Recruitment

Data were scraped from the Reddit website, a social media platform where anonymous users may post and interact with content organized into certain communities termed “subreddits.” We examined subreddits centered around COVID-19–related discussions and were likely to have uncivil discourse. For instance, one subreddit of interest was r/nonewnormal, a place for people to discuss and criticize COVID-19 lifestyle disruptions. R/nonewnormal would have been a worthwhile subreddit to scrape data from, but because it was banned for its strong antivaccine and antimask content, we could not collect the data [59]. Because we expected users who frequented r/nonewnormal to move and become active in other subreddits, we leveraged the Subreddit Stats website [60,61]. This website provides statistics on various subreddits and the relationships among them. Two subreddits were identified as having a large user overlap with r/nonewnormal, namely, r/coronaviruscirclejerk and r/lockdownskepticism [62]. The 2 subreddits were selected because of their large overlap with the r/nonewnormal subreddit. The subreddit r/coronaviruscirclejerk primarily included satirical posts ridiculing others who worry about COVID-19, whereas the subreddit r/lockdownskepticism focused on a more empirical discussion that questioned the actual effectiveness of pandemic lockdowns and quarantines. Both subreddits, similar to r/nonewnormal, were illuminating given their focus on COVID-19–related discussion and the high likelihood of them containing uncivil discourse.

**Figure 1:**
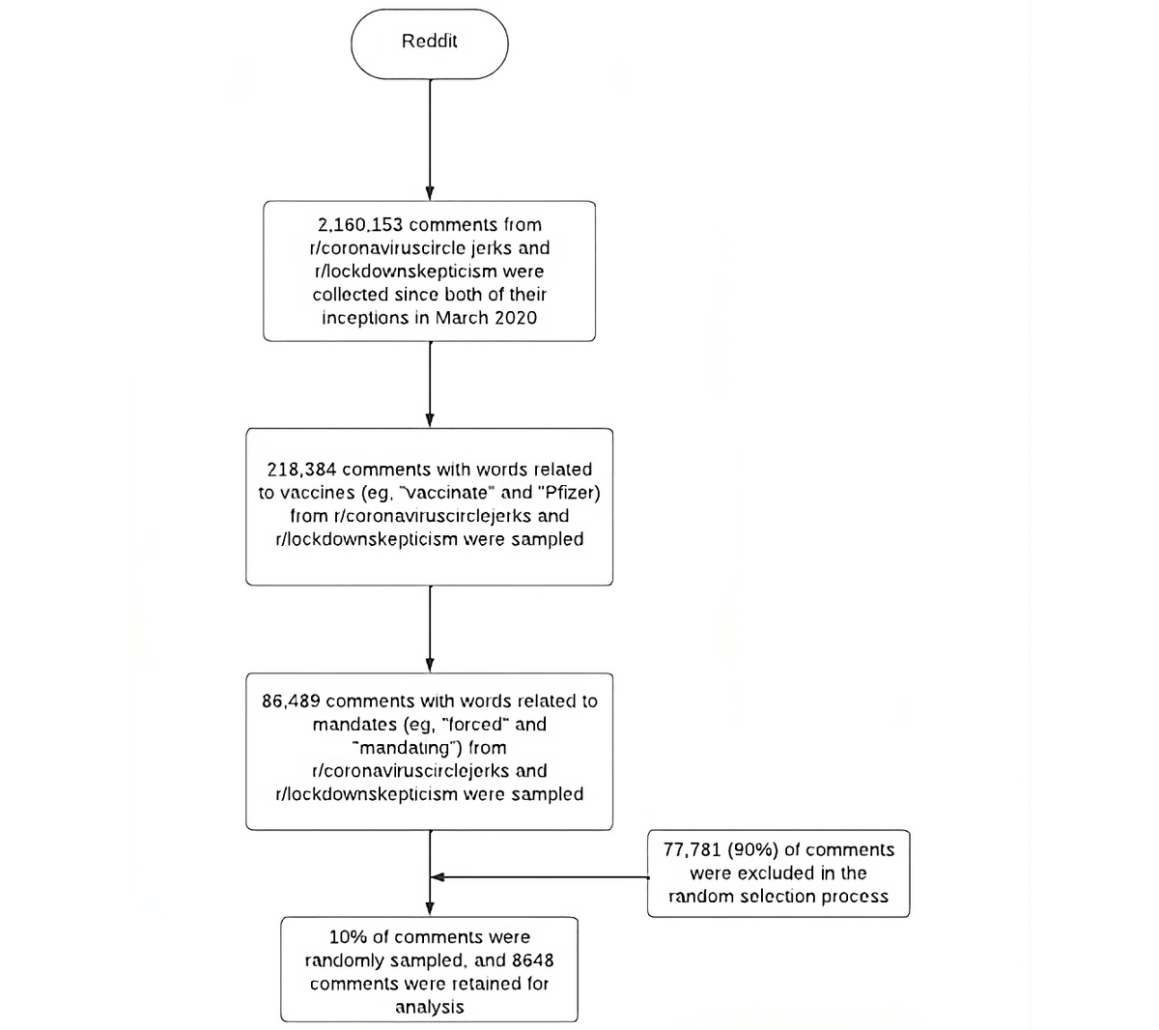
Data collection and filtration process flowchart.

All posts and comments from r/coronaviruscirclejerk and r/lockdownskepticism were collected since their inception in March 2022. The collected data set was then filtered for key terms related to words related to the COVID-19 vaccine (eg, “vaccinate” and “Pfizer”) and key terms related to mandates (eg, “forced” and “mandating”; see Textboxes 1 and 2 for details). The research team took a grounded theory approach to key term selection, including a systematic review of existing literature and popular press for relevant terms (see Figure 1 for details). In addition, the terms were selected based on their salience in the r/coronaviruscirclejerk and r/lockdownskepticism subreddits. A 10% random sample of the comments that contained vaccine and mandate terms was retained for analysis.

**Keywords for vaccines.**

**Vaccine keywords**

dose, johnson, J&J, Jnj, pfizer, moderna, covax, vax, vaccine, vaccinate, Vaccinated, vaccinates, vaccinating, needle, inject, injected, injecting, inoculated, inoculates, inoculating, immunization, immunity, immune, shot, shots, jab, jabbed, jabs, booster, boosted, sputnik, mRNA, comirnaty, spikevax, astrazeneca, covishield, vaxzeveria, janssen, coronavac, epivac, epivaccine, convidicea, unvaxed, unvaxxed, unvaccinated, biontech, az, sinopharm, sinovac, covovax, nuvaxovid

**Keywords for mandates.**

**Mandate keywords**

mandate, mandating, mandated, mandates, force, forcing, forced, forces, require, required, requiring, requires, make, making, made, Makes, coerce, coercing, coerced, coerces, must, need, needing, needed, needs, order, ordering, orders, ordered, necessitate, necessitating, Necessitates, necessitated, demand, demands, demanded, demanding, instruct, instructed, instructing, instructs, command, commanding, commanded, commands, freedom, freedoms, liberties, violate, violating, violated, violates, right, rights

### Statistical Analyses

Data analysis was a 2-pronged approach. We first examined the presence and nature of incivility in comments and then identified the presence of any discussion of the 5 moral foundations in each comment. Perspective application programming interface (API), a tool that uses machine learning to detect comment incivility, was used to measure 5 dimensions of incivility, namely, toxicity, insults, profanity, threat, and identity attack (see Textbox 3 for definitions and examples). Perspective API is a validated tool trained by human coders on large data sets with millions of comments and has been used in studies across various contexts, such as health and politics [17,20,63,64]. Perspective API assigns each comment a score from 0 to 1 per dimension of incivility based on how closely the comment reflected the specific dimension and how likely it was to impact a conversation. To determine the moral foundations reflected in each comment, we used a prevalidated MFT dictionary [44]. This dictionary tool generated the relative frequency of words associated with each moral foundation as a score from 0 to 1. Similar studies have used prevalidated count dictionaries to measure the aspects of COVID-19 discourse [5,65].

**Description of the moral foundations.**

**Sanctity and degradation**

- Characterized by concerns for physical and spiritual purity, along with chastity [30]

**Care and harm**

- Characterized by motivations to protect and care against suffering [29]

**Fairness and cheating**

- Characterized by concerns against unfairness, cheating, and inequities [29,30]

**Loyalty and betrayal**

- Characterized by devotion and allegiance to a group and loyalty [30]

**Authority and subversion**

- Characterized by obedience, respect, and fulfillment of obligations to hierarchical relationships [30]

### Ethical Considerations

This study does not include any personally identifiable information and only relies on publicly available data. The institutional review board recognizes that the analysis of publicly available data does not fall under human subject research. As such, ethical review and approval were not required for this study.

## Results

A Spearman correlation analysis was conducted to measure the association between the 5 different dimensions of incivility and the 5 different moral foundations (see Table 1).

**Table 1:** Five dimensions of comment incivility^a^.

Dimension	Perspective API^b^ definition	Example comments
Toxicity	“A rude, disrespectful, or unreasonable comment that is likely to make people leave a discussion.”	“Don’t they realize that Dee Snider (Twisted Sister) loves vaccine mandates and hates anti vax. Fuck that asshole.”
Insults	“Insulting, inflammatory, or negative comment towards a person or a group of people.”	“You’re the one calling for your coworkers to burn in hell for requiring basic safety for one another. Vaccine mandates aren’t ‘communist’, they’ve been around longer than the United States. You’re just a selfish prick with their head up ass.”
Profanity	“Swear words, curse words, or other obscene or profane language.”	“Fucking Disneyland isn't enforcing masks anymore there is no fucking reason for your college to do so especially since they are requiring the vaccine.”
Threat	“Describes an intention to inflict pain, injury, or violence against an individual or group.”	“If they come to your house and try to force vaccine on you or your family members, just take knife, scissors, axe, or something really sharp and... you know what to do. Even if they kill you after that, your life will have more meaning than if you would comply. It’s sad that we have to talk about this, but there we are.”
Identity attack	“Negative or hateful comments targeting someone because of their identity.”	“You are wrong. The elders are outside protesting. The youth is having gay orgies in clubs and protesting against capitalism but don’t care about forced vaccinations.”

^a^Dimensions of incivility were measured via Perspective API [66].

^b^API: application programming interface.

A significant positive correlation was observed between purity and each of the 5 dimensions of incivility: identity attack (*P*<.001; 95% CI 0.07-0.11), insult (*P*<.001; 95% CI 0.11-0.16), toxicity (*P*<.001; 95% CI 0.10-0.14), profanity (*P*<.001; 95% CI 0.07-0.11), and threat (*P*<.001; 95% CI 0.05-0.09). A significant positive correlation was observed between fairness and the dimensions of identity attack (*P*<.001; 95% CI 0.08-0.12), insult (*P*<.001; 95% CI 0.05-0.09), toxicity (*P*<.001; 95% CI 0.04-0.08), and profanity (*P*<.001; 95% CI 0.02-0.07). A significant positive correlation was observed between harm and the dimensions of identity attack (*P*=.003; 95% CI 0.02-0.06), insult (*P*<.001; 95% CI 0.04-0.08), toxicity (*P*<.001; 95% CI 0.04-0.08), and threat (*P*<.001; 95% CI 0.05-0.10). A significant positive correlation was observed only between in-group loyalty and identity attack (*P*=.009; 95% CI 0.02-0.06). There were no significant correlations between authority and any of the dimensions of incivility (see Table 2). In addition, the descriptive results for incivility by subreddit and moral foundations by subreddit are included below (see Tables 3 and 4).

**Table 2:** Spearman correlation results among dimensions of incivility and moral foundations (N=8648).

Moral foundation	Identity attack		Insult			Toxicity			Profanity			Threat	
	ρ	*P* value^a^	ρ	*P* value^a^	ρ		*P* value^a^	ρ		*P* value^a^	ρ		*P* value^a^
Purity	0.09	<.001	0.13	<.001	0.12		<.001	0.09		<.001	0.07		<.001
Fairness	0.10	<.001	0.07	<.001	0.06		<.001	0.05		<.001	0.02		.62
Harm	0.05	.003	0.06	<.001	0.06		<.001	0.03		.18	0.08		<.001
In-group loyalty	0.04	.009	0.02	>.99	0.00		>.99	−0.02		>.99	−0.02		.81
Authority	0.00	>.99	0.01	>.99	0.00		>.99	−0.02		.45	−0.01		>.99

^a^*P* values were adjusted using the Holm correction.

**Table 3:** Summary statistics table for dimensions of incivility by subreddit^a^.

Variable			Value, mean (SD)		Value, n		Value, SE		Value, median (range)
**Identity attack**									
	r/lockdownskepticism	0.10 (0.11)		4961		0.002		0.06 (0.00004-0.94)	
	r/coronaviruscirclejerk	0.13 (0.14)		3687		0.002		0.08 (0.0007-0.97)	
**Threat**									
	r/lockdownskepticism	0.18 (0.19)		4961		0.003		0.10 (0.0007-0.98)	
	r/coronaviruscirclejerk	0.23 (0.22)		3687		0.004		0.13 (0.006-0.99)	
**Insult**									
	r/lockdownskepticism	0.16 (0.20)		4961		0.003		0.08 (0.0004-0.98)	
	r/coronaviruscirclejerk	0.20 (0.23)		3687		0.004		0.11 (0.002-0.98)	
**Toxicity**									
	r/lockdownskepticism	0.19 (0.21)		4961		0.003		0.10 (0.0004-0.99)	
	r/coronaviruscirclejerk	0.24 (0.25)		3687		0.004		0.13 (0.004-0.99)	
**Profanity**									
	r/lockdownskepticism	0.11 (0.21)		4961		0.003		0.04 (0.0002-0.98)	
	r/coronaviruscirclejerk	0.15 (0.25)		3687		0.004		0.05 (0.001-0.99)	

^a^The Google Perspective application programming interface was used to measure dimensions of incivility.

**Table 4:** Summary statistics table for moral foundations by subreddit^a^.

Variable		Value, mean (SD)	Value, n	Value, SE	Value, median (range)
**Harm**					
	r/lockdownskepticism	0.34 (1.36)	4961	0.02	0.00 (0.00-15.38)
	r/coronaviruscirclejerk	0.43 (1.67)	3687	0.03	0.00 (0.00-22.22)
**Fairness**					
	r/lockdownskepticism	0.23 (0.14)	4961	0.02	0.00 (0.00-18.18)
	r/coronaviruscirclejerk	0.18 (1.05)	3687	0.02	0.00 (0.00-16.67)
**Authority**					
	r/lockdownskepticism	0.37 (1.36)	4961	0.02	0.00 (0.00-20.00)
	r/coronaviruscirclejerk	0.37 (1.46)	3687	0.02	0.00 (0.00-16.67)
**In-group**					
	r/lockdownskepticism	0.13 (0.87)	4961	0.01	0.00 (0.00-20.00)
	r/coronaviruscirclejerk	0.14 (0.94)	3687	0.02	0.00 (0.00-18.75)
**Purity**					
	r/lockdownskepticism	0.13 (0.87)	4961	0.01	0.00 (0.00-16.67)
	r/coronaviruscirclejerk	0.18 (1.12)	3687	0.02	0.00 (0.00-16.67)

^a^A prevalidated moral foundation dictionary was used to assess the moral foundations of the posts through a computer-assisted text scanner.

A 2-tailed independent samples *z* test was conducted to examine whether the mean of identity attack, insult, toxicity, profanity, and threat was different in the r/lockdownskepticism and r/coronaviruscirclejerk subreddits. Results were significant for all 5 dimensions of incivility (*P*<.001), indicating that the null hypotheses can be rejected (see Table 5 for details). This suggests that the mean of identity attack, insult, toxicity, profanity, and threat in the r/lockdownskepticism subreddit was significantly lower than that in the r/coronaviruscirclejerk subreddit.

**Table 5:** Two-tailed independent samples z test for incivility by subreddit (N=8648).

Variable	r/lockdownskepticism, mean (SD)	r/coronaviruscirclejerk, mean (SD)	*z*	*P* value
Toxicity	0.19 (0.21)	0.24 (0.25)	−10.33	<.001
Identity attack	0.10 (0.11)	0.13 (0.14)	−9.32	<.001
Threat	0.18 (0.19)	0.23 (0.22)	−10.55	<.001
Insult	0.16 (0.20)	0.20 (0.23)	−8.82	<.001
Profanity	0.11 (0.21)	0.15 (0.25)	−7.91	<.001

## Discussion

### Principal Findings

Understanding incivility surrounding public health initiatives, such as COVID-19 vaccine mandates, is imperative to improve public health efforts and public well-being. This work revealed differing associations between the moral foundations and each dimension of comment incivility.

First, as purity increased, so did all 5 dimensions of comment incivility, supporting hypothesis H1 as expected. The following finding is consistent with the study by Frimer et al [39], concluding that purity has been an area of political disagreement. The association between purity and incivility also supports the idea that there may indeed be 2 sides to purity and can explain why purity can predict both COVID-19 protective behaviors and vaccine hesitancy, as seen in recent research [32]. In addition, according to Amin et al [37], current provaccine messaging has been created with a focus on the harm and fairness foundations. However, public health professionals should consider designing messages appealing to other moral foundations, like purity. For example, messages could be designed keeping in mind the domain words associated with the moral foundation (eg, “Getting a vaccine can help your body fight against the impure COVID-19 virus”) [44].

Second, as fairness increased, all dimensions of comment incivility except for comment threat tended to increase, partially supporting hypothesis H2. Fairness did not predict comment threat, which may be, in part, due to the political polarization associated with moral foundations and COVID-19. In a research report, Harward et al [67] provide an example of how conservative individuals have viewed fairness in society as a “get what you deserve” system instead of a system of equity. It is possible that some conservative individuals may not have felt prompted to make threats against vaccine proponents because they believe proponents have already posed a threat to themselves by getting an “impure” vaccine. This finding implies that COVID-19 vaccine incivility is influenced heavily by the purity foundation, but in this context, it manifests as a fairness concern. This finding increases the need for public health officials to investigate how valuing purity relates to vaccine uptake and, consequently, tailor messaging to address vaccine impurity concerns.

Third, as harm increased, all dimensions of comment incivility except for comment profanity tended to increase, partially supporting hypothesis H3. One potential explanation for this is a finding from Feldman et al [68] that notes a positive relationship between profanity and honesty. Individuals may have been dishonest when engaging in uncivil discussion involving the care and harm foundation. Additionally, it is possible that although there is some incivility correlated with harm, it may be a facade to hide one’s genuine concerns with COVID-19 vaccine mandates. An implication of this is that public health officials may have been misled by what individuals were concerned about regarding COVID-19 vaccine mandates. This offers one explanation for the prevalence of existing provaccine messaging that appeals to the value of harm [37]. Public health officials should be mindful of the possibility that concerns of fairness surrounding the COVID-19 vaccine mandates may not be as prominent as observed.

Fourth, as in-group loyalty increased, only comment identity attack tended to increase, partially supporting hypothesis H4. One study found no association between the amount of “exclusionary language” used and comment toxicity [69]. Also, Brewer [70] notes that out-group hostility may also have resulted from a desire to gain political power, a possibility given that COVID-19 has been politicized [3]. This implies that a small part of the uncivil COVID-19 discourse may be due to an issue of in-group loyalty. To address this concern and increase vaccination numbers, public health officials and lawmakers should find a way to bridge partisan gaps in the United States.

Fifth, there was no correlation between authority and any of the dimensions of incivility, refuting hypothesis H5. One possible reason is that discussion about COVID-19 vaccine mandates may be more of a political in-group concern rather than a concern of authority, given that health information has been heavily politicized [3,71]. Although contrary to our hypothesis, this result still has important implications. For instance, these findings suggest that uncivil discourse regarding COVID-19 vaccine mandates has been an issue of policy and preservation rather than an issue of the policy makers and policy endorsers. In other words, the incivility surrounding COVID-19 vaccine mandates has little to do with authority figures such as the CDC, the president, or local public health authorities. This suggests that public health efforts may not have been affected by the reputation of authority figures but that they are affected by the way public health policies are perceived.

Further, we investigated whether incivility varies by platform norms. The findings revealed that the means of the different dimensions of incivility (eg, toxicity, severe toxicity, insult, profanity, threat, and identity attacks) in the r/lockdownskepticism subreddit were significantly lower than those in the r/coronaviruscirclejerk subreddit, suggesting that incivility does vary by platform norms. Recent studies have argued that platform norms vary within web-based cultures and within the microcultures of these platforms [17]. Because Reddit moderator roles differ for each subreddit, our results point toward variations within Reddit in discourse incivility. Simply put, discourse incivility can vary within Reddit as moderators have different rules that can impede or foster incivility. These results are consistent with prior research that has pointed to variations in incivility by platform [56,57,72].

### Practical Implications

The COVID-19 pandemic has caused disruptions in all facets of life worldwide. Therefore, restoring trust in public health agencies and protocols is paramount. Tailored public health messaging incorporating social values (eg, moral foundations) may help to reach, educate, and persuade individuals in a way that evokes civil responses and improve compliance [73-76]. One way health messages can be tailored is by addressing the moral values underlying the concerns people may have about public health interventions (eg, vaccinations and screenings), which could manifest as uncivil discourse. Existing literature has argued that developing customized messaging based on moral foundations can be effective in persuading individuals. Specifically, scholars have found that reframing messages based on moral foundations congruent to individual attitudes can persuade conservatives and liberals to agree on environmental issues [77] and enhance participation in sustainable environmental practices [78]. Other studies have found that issues framed using moral foundations can not only strengthen existing attitudes but also shift attitudes among liberals and conservatives [79]. Another approach can be to customize public health messages based on the levels of incivility observed in different areas of social media platforms. For instance, in our study, we found that the r/coronaviruscirclejerk subreddit had higher levels of different dimensions of incivility than r/lockdownskepticism. There is also evidence that Twitter is less toxic than Facebook as more uncivil comments are found on Facebook [56,57]. Given these platform-based cultural differences, it may be beneficial for health care professionals and lawmakers to develop messages targeted at these differences. Specifically, these messages could be directed toward areas within a platform (ie, Reddit and Facebook) where incivility is more salient. Ultimately, we argue that the antecedents of incivility can inform public health interventions. Existing studies have found a link between incivility and negative emotions such as sadness and anxiety [20]. Therefore, public health messaging that evokes such emotions could result in uncivil discourse from the public. However, the underlying mechanisms fueling these emotional reactions remain unclear. By considering the moral foundations fueling incivility, public health officials can design effective messages aimed at appealing to specific moral foundations that will, in turn, increase vaccine uptake and overall community well-being.

### Limitations

This study is novel in that it uses a data set comprising posts from users who are banned from the antivaccine subreddits, which sheds light on unique and otherwise unexplored discourse. Another strength of our research is that it focuses on Reddit, a platform that is often overshadowed by Facebook and Twitter in extant literature. However, our study is also limited in that it focused on 2 specific subreddits, which are not representative of all vaccine-related discourse on Reddit and other social media. Future studies may benefit by focusing on comparing vaccine-related discourse across different social media platforms with a more comprehensive data set. Another limitation of our research is its observational nature that prevents us from implying causation. An extension of this study could focus on establishing a causal relationship between moral foundations and COVID-19 vaccine mandate incivility, if such a relationship exists. Also, our study used only 10% of the sample because of scarce computational resources, which limited us in our analyses. Future researchers should also consider investigating how moral foundations interact with different dimensions of incivility with a larger data set. In addition, future research can also use qualitative approaches and interview users who post uncivil conduct on social media sites such as Reddit. Because incivility is a multifaceted construct, such analyses can aid our understanding of incivility and shed light on the psychological processes that lead to uncivil discourse.

### Conclusions

**Figure 2:**
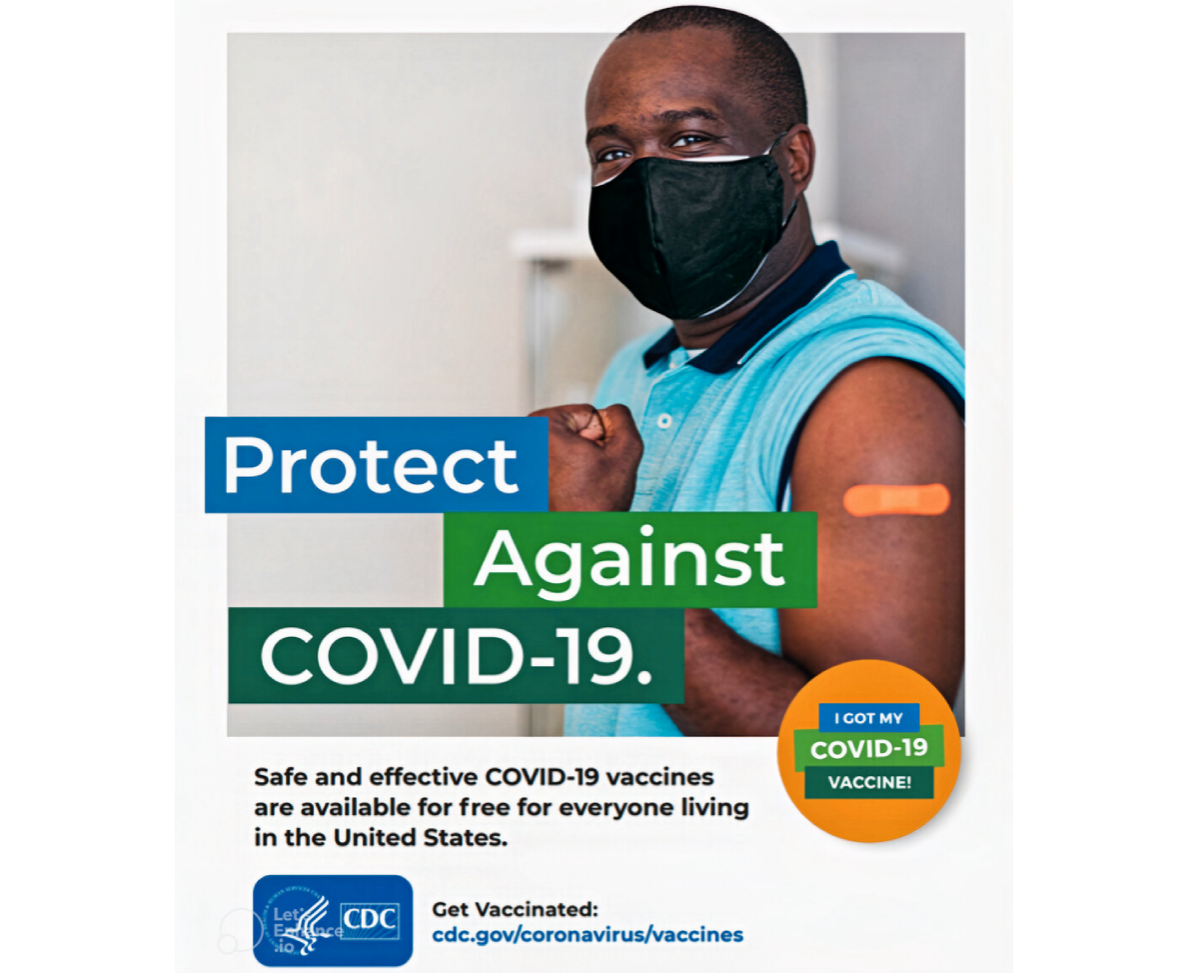
Centers for Disease Control and Prevention (CDC) messaging highlighting the care and fairness moral foundations.

This paper examined whether moral foundations were related to incivility surrounding COVID-19 vaccine discourse. We found that purity, fairness, harm, and in-group loyalty were positively related to different dimensions of incivility. This study adds to the growing literature focused on theorizing the mechanisms behind incivility related to COVID-19–related discourse. Our findings highlight the need for health campaigns to design messages appealing to specific moral foundations of specific demographics. For example, organizations such as the CDC have already created messaging that highlights moral foundations such as care and fairness by highlighting the protective nature of vaccines and their availability for all individuals (see Figure 2 for details). By integrating moral foundations, messaging related to COVID-19 may be an effective way to persuade audiences to follow public health protocols and engage in civil discourse.

## Abbreviations

API: application programming interface
CDC: Centers for Disease Control and Prevention
LGBTQ: lesbian, gay, bisexual, transgender, queer
MFT: moral foundations theory
RQ: research question

## Data Availability

The data sets generated during and/or analyzed during this study are available from the corresponding author on reasonable request.
